# Analysis of Multi-Site Bile Acid Metabolic Profile Reveals an Association Between Bile Acid–FXR Signaling and Visceral Hypersensitivity in Heterotypic Intermittent Stress-Induced IBS

**DOI:** 10.3390/ijms27209293

**Published:** 2026-10-19

**Authors:** Shiyuan Jiang, Chang Gao, Yafang Song, Ninan Zhang, Zhiyun Zhang, Xiaoyu Liu, Yangshuai Su, Xiaoyu Wang, Tao Li

**Affiliations:** 1 Institute of Acupuncture and Moxibustion, China Academy of Chinese Medical Sciences, Beijing 100700, China; 2 Acupuncture-Moxibustion and Tuina College, Nanjing University of Chinese Medicine, Nanjing 210023, China; 3 Experimental Research Center, China Academy of Chinese Medical Sciences, Beijing 100700, China

**Keywords:** irritable bowel syndrome, visceral hypersensitivity, bile acids, farnesoid X receptor, heterotypic intermittent stress, HPLC-MS/MS, calcitonin gene-related peptide

## Abstract

Visceral hypersensitivity (VH) is a hallmark of irritable bowel syndrome (IBS), establishing a reliable VH model is vital for investigating the disorder. While the impact of chronic stress on bile acid (BA) metabolism remains unclear. The exploratory study aimed to evaluate a heterotypic intermittent stress (HIS)-induced rat model of VH and characterize associated alterations in BA metabolism and farnesoid X receptor (FXR) signaling. Rats underwent a 9-day HIS regimen, and VH was assessed by electromyographic recording of the visceromotor response to colorectal distension. BA profiles in feces, colon, and serum were quantified by HPLC-MS/MS, and colonic FXR and CGRP expression were examined by Western blot, immunohistochemistry, and three-dimensional confocal reconstruction. HIS induced significant VH without organic colonic pathology, accompanied by increased CGRP-positive nerve fibers. The bile acid pool composition region-specific alterations in feces, colon, and serum. Specifically, the free-to-conjugated BA ratio was significantly increased in the colon and decreased in the serum of HIS rats, whereas no significant difference was detected in feces, while species-level changes were compartment-specific. Colonic FXR was upregulated and spatially associated with CGRP-positive fibers; in a preventive guggulsterone-treated group, both signals were reduced. These findings indicate that HIS-induced VH is associated with region-specific alterations in BA metabolism and colonic FXR signaling change, identifying the BA–FXR axis as a candidate pathway meriting functional validation as a potential therapeutic target for IBS.

## 1. Introduction

Irritable bowel syndrome (IBS) is a prevalent functional gastrointestinal disorder affecting approximately 5–10% of the general population [1], primarily characterized by symptoms of discomfort such as bloating [2], abdominal pain, and irregular bowel movements, with pathogenesis involving gut microbiota dysbiosis [3], visceral afferent dysfunction [4], and local immune activation [5]. Visceral hypersensitivity (VH), an enhanced responsiveness of visceral tissues to mechanical and chemical stimuli, serves as a crucial indicator for IBS diagnosis and evaluation [6]. VH is intricately linked with triggering factors such as post-infection inflammation and emotional or environmental stress [7], which are principal contributors to the bloating and abdominal pain symptoms experienced by individuals with IBS. Consequently, the development of a reliable and effective model of VH is fundamental for investigating the biological mechanisms underlying this condition. Although several studies have demonstrated that drug enemas such as TNBS [8], capsaicin and acetic acid [9] can induce VH, their utility is limited by drug-related adverse effects and the prediction errors of single models [10]. Experimental models based on stress—whether acute stress models or chronic stress models [11]—can more accurately replicate the key clinical characteristics of IBS and are therefore more suitable for mechanistic studies.

Bile acid (BA) represents the terminal metabolites of cholesterol. The liver metabolizes cholesterol, converting it into primary BAs. Subsequently, these primary BAs undergo transformation into secondary BAs within the intestine, facilitated by the enzyme bile salt hydrolase (BSH). BAs are reabsorbed into the liver via the intestines, thereby perpetuating the enterohepatic circulation [12]. As steroids with amphiphilic characteristics and essential components of bile [13], BA plays significant roles in metabolism, exerts antibacterial effects, and facilitates lipid dissolution. Current research indicates that aberrant BA metabolism significantly contributes to IBS pathogenesis and VH induction [14]. Clinical investigations have demonstrated that approximately 30% of irritable bowel syndrome with diarrhea (IBS-D) patients exhibit BA malabsorption [15]. Experimental studies in animal models have indicated that the acute administration of deoxycholic acid (DCA) into the colon of rats results in heightened colonic sensitivity to stimuli and promotes the development of VH [6]. Moreover, BA interacts with the bile acid-sensitive nuclear receptor farnesoid X receptor (FXR) to modulate BA homeostasis and contribute to VH [16]. Despite these findings, the pathological mechanisms underlying BA metabolism and receptor expression in IBS remain inadequately understood.

Therefore, this study aimed to validate a heterotypic intermittent stress (HIS)-induced rat model of VH and to characterize BA metabolic changes across feces, colon, and serum. This research used high-performance liquid chromatography–mass spectrometry (HPLC-MS/MS) analysis of BA metabolism with molecular detection and 3D imaging of FXR and calcitonin gene-related peptide (CGRP)-positive nerve fibers technology and aims to elucidate the role of the BA metabolism and FXR signaling in stress-induced VH, providing novel insights and methodological references for research into the mechanisms of IBS.

## 2. Results

### 2.1. HIS-Induced VH in IBS Rats

**Figure 1:**
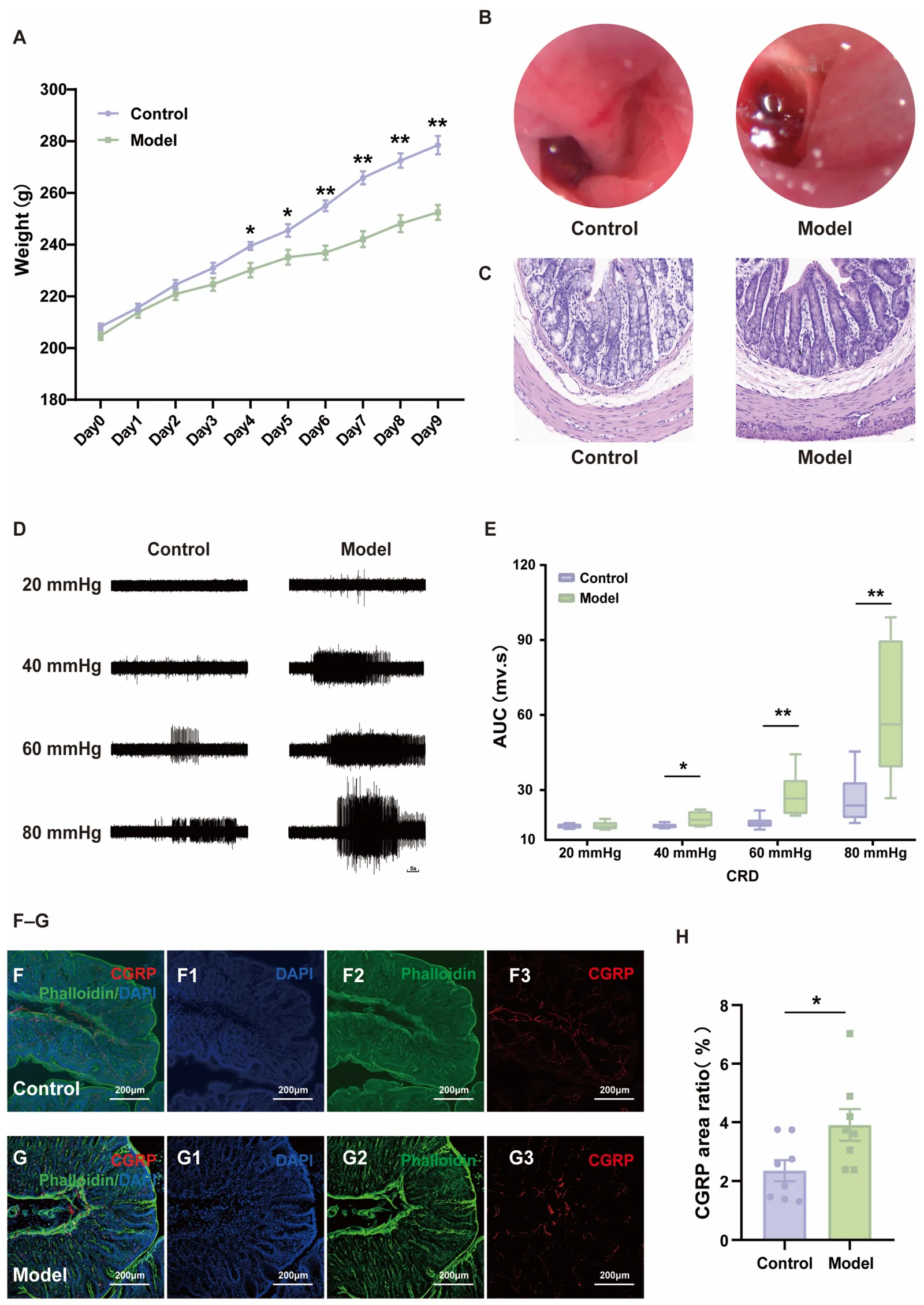
Effects of HIS on VH and CGRP-positive nerve fibers in IBS rats. (**A**) Body weight changes of rats in the control and model groups during the 9-day HIS intervention. (**B**) Representative colonoscopic images of the colonic mucosa in the control and model groups. (**C**) Representative HE-stained sections of colonic tissues from the control and model groups. (**D**) Representative EMG schematic of abdominal wall muscle responses to CRD at pressures of 20, 40, 60, and 80 mmHg in both groups. (**E**) Quantitative analysis of the AUC of CRD-induced visceral motor responses. (**F**,**G**) Representative IF staining images of colonic tissues from control (**F**) and model (**G**) rats, showing merged staining of CGRP (red), Phalloidin (green), and DAPI (blue); individual channels are shown for DAPI (**F1**,**G1**), Phalloidin (**F2**,**G2**), and CGRP-positive nerve fibers (**F3**,**G3**). Scale bar = 200 µm. (**H**) Quantification analysis of the CGRP-positive nerve fiber area ratio. Data are expressed as mean ± SEM (*n* = 8). * *p* < 0.05, ** *p* < 0.01.

After 9 days of HIS intervention, the body weight of model rats was significantly reduced compared to that of controls (*n* = 8; * *p* < 0.05, ** *p* < 0.01) (Figure 1A), while colonoscopy and hematoxylin–eosin (HE) staining revealed intact colonic mucosa with regularly arranged crypts and no congestion, edema, ulceration, or inflammatory infiltration (Figure 1B,C), consistent with the absence of organic lesions characteristic of IBS. To assess the potential for VH induced by HIS, electromyography (EMG) was employed to evaluate visceral sensitization in both model and control group rats. Following colorectal distension (CRD) stimulation of the rat colon at pressures of 20, 40, 60, and 80 mmHg, the area under the curve (AUC) values for VH in the control group were 15.509 ± 0.315, 15.728 ± 0.316, 16.971 ± 0.833, and 26.381 ± 3.394 mV·s, respectively. In contrast, the model group exhibited significantly elevated AUC values of 15.776 ± 0.567, 18.395 ± 1.013, 27.992 ± 3.064, and 60.677 ± 9.372 mV·s (*n* = 8; * *p* < 0.05, ** *p* < 0.01) (Figure 1D,E). Moreover, multiplex immunofluorescence (IF) staining of CGRP, phalloidin and DAPI revealed, compared to the control group, a significantly increased area ratio of CGRP-positive nerve fibers in the colon of model rats (*n* = 8; * *p* < 0.05) (Figure 1F–H). This suggests that the potential mechanism underlying HIS-induced VH is CGRP-mediated enhancement of sensory innervation. These findings validate that HIS elicits robust VH without inducing organic colonic injury, a phenotype accompanied by augmented CGRP-expressing sensory innervation.

### 2.2. Effects of HIS on BA Metabolism in IBS Rats

**Figure 2:**
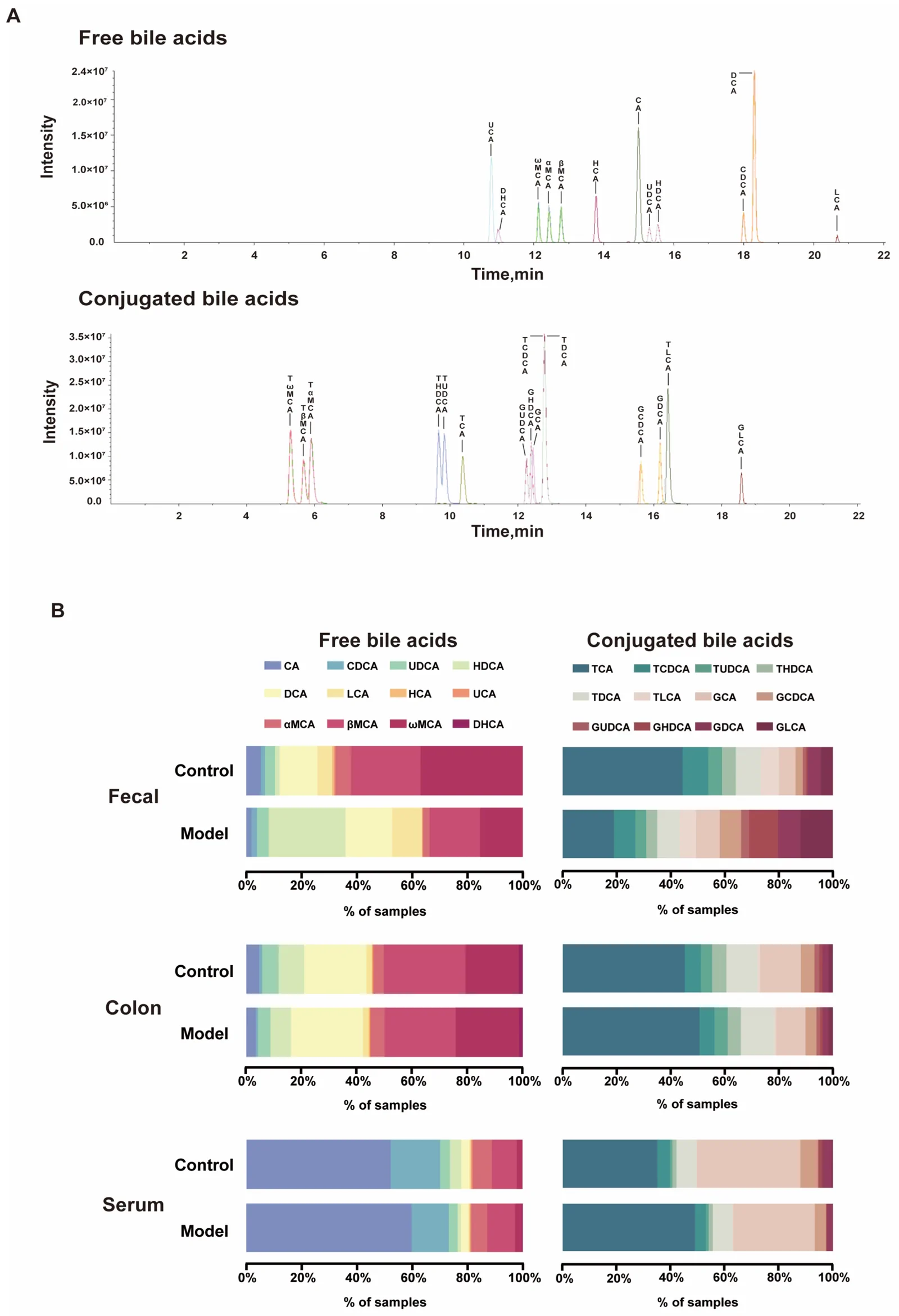
Effects of HIS on BA composition in the feces, colon, and serum of control and model rats. (**A**) Representative HPLC-MS/MS chromatograms depicting standards of free and conjugated BAs. (**B**) The abundance and composition of free and conjugated BAs in the feces, colon, and serum of control and model rats. Among the 27 endogenous BAs initially screened, 24 were included in the final composition analysis; three tauro-muricholic acids (T-α-MCA, T-β-MCA, and T-ω-MCA) were excluded due to insufficient detection (*n* = 8).

To investigate whether HIS affects BA metabolism, BA profiles in the feces, colon, and serum of rats were quantified by HPLC-MS/MS. Representative chromatograms of authentic BA standards are shown in Figure 2A, and BAs were classified as free or conjugated according to their chemical structures. The influence of HIS on BA metabolism was assessed by comparing BA concentrations across the groups. Overall, the BA content was highest in feces, followed by the colon and serum, with free BAs predominant. However, the effect of HIS on the free-to-conjugated bile acid ratio was tissue-specific. In feces, no statistically significant difference in the free-to-conjugated ratio was detected between the control and model groups (*n* = 8; NS > 0.05); this nonsignificant result should not be interpreted as evidence of equivalence. The colonic free-to-conjugated ratio and free BAs fraction were significantly increased in the model group compared with the control (*n* = 8; ** *p* < 0.01), accompanied by a significant decrease in the conjugated BAs fraction (*n* = 8; ** *p* < 0.01). Conversely, in serum, the free-to-conjugated ratio and free BAs fraction of the model group were significantly decreased compared with the control (*n* = 8; ** *p* < 0.01), with a significant increase in the conjugated BAs fraction (*n* = 8; ** *p* < 0.01). Moreover, the relative abundance of individual BA species was markedly changed (Figure 2 and Table 1). These findings indicate that HIS intervention alters BA conjugation and distribution in a compartment-specific manner.

Among the 27 endogenous BAs initially screened, 24 were included in the final composition analysis; three tauro-muricholic acids (T-α-MCA, T-β-MCA, and T-ω-MCA) were excluded due to insufficient detection. The 24 BAs analyzed are listed in Figure 2B. Among free BAs (Figure 2B), ωMCA (36.94%), βMCA (25.22%), and DCA (13.63%) were the main fecal species in control rats, whereas HDCA (27.81%), βMCA (18.26%), and DCA (16.83%) predominated in model rats. In the colons of both control and model groups, the percentages of βMCA (29.52%/25.66%), DCA (22.50%/25.96%), and ωMCA (19.30%/22.99%) predominated, and serum was predominated by CA (52.06%/59.81%), CDCA (17.92%/13.42%), and βMCA (8.99%/10.04%). Among conjugated BAs, TCA (44.40%), TCDCA (9.48%), and TDCA (9.27%) were most abundant in the feces of control rats, whereas TCA (18.97%), GLCA (12.03%), and GHDCA (10.74%) predominated in the model group. In the colon, TCA (45.16%/50.62%), GCA (15.12%/10.95%), and TDCA (11.56%/12.21%) were the major conjugated species in both groups, while serum was mainly of GCA (38.36%/30.22%), TCA (35.00%/48.95%), and TDCA (7.39%/7.46%) (*n* = 8). These results indicate that HIS remodelled the composition of the BA pool in IBS rats without changing the overall free-to-conjugated ratio.

BA concentrations were quantified by HPLC-MS/MS and are expressed as mean ± SEM in ng/g (fecal and colon samples) or ng/mL (serum samples); For each animal, free and conjugated BAs concentrations were measured in colon and serum. The free fraction (%) was calculated as

$$\mathrm{free fraction}=\frac{\left[\mathrm{free}\right]}{\left[\mathrm{total}\right]}\times 100\%$$

The conjugated fraction (%) was calculated as

$$\mathrm{Conjugated fraction}=\frac{\left[\mathrm{conjugated}\right]}{\left[\mathrm{total}\right]}\times 100\%$$

The free-to-conjugated ratio was calculated as:

$$\mathrm{Free}/\mathrm{conjugated ratio}=\frac{\left[\mathrm{free}\right]}{\left[\mathrm{conjugated}\right]}$$

### 2.3. The Impact of HIS on BA Levels in the Feces, Colon, and Blood Serum of Rats with IBS-Related VH

**Figure 3:**
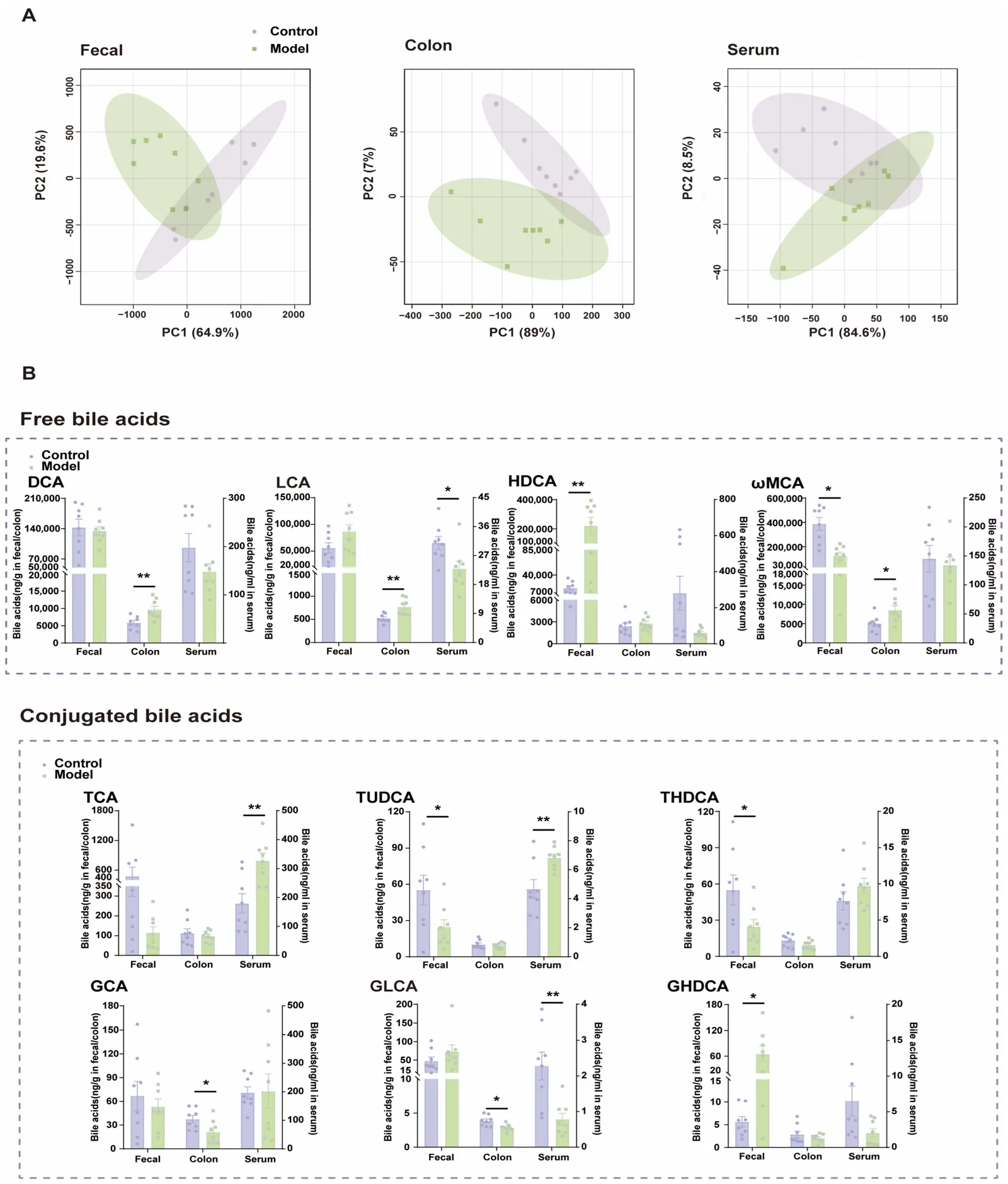
Effects of HIS onBA concentrations in the feces, colon, and serum of control and model rats. (**A**) PCA was employed to assess BAs in the feces, colon, and serum of both control and model rats (*n* = 8). (**B**) Quantification of individual BAs by HPLC-MS/MS. Among the 27 endogenous BAs initially screened, 24 were included in the final composition analysis; three tauro-muricholic acids (T-α-MCA, T-β-MCA, and T-ω-MCA) were excluded due to insufficient detection. Data are expressed as mean ± SEM. (*n* = 8); * *p* < 0.05, ** *p* < 0.01.

Principal component analysis (PCA) was employed to identify the similarities and variations in BA composition across fecal, colonic, and serum samples from both control and model groups. The analysis revealed a distinct angular difference between the model and control groups, suggesting alterations in the BA profile of rats within the model group (Figure 3A). To assess the impact of HIS on BA metabolism, BA levels in fecal, colonic, and serum samples were compared between normal rats and those with IBS-induced VH. Post-HIS intervention, an increase in fecal HDCA and GHDCA levels was observed, whereas ωMCA, TUDCA, and THDCA levels decreased. In the colon, DCA, LCA, and ωMCA levels rose, while GCA and GLCA levels declined. In serum, LCA and GLCA levels decreased, whereas TCA and TUDCA levels increased (*n* = 8, ** *p* < 0.01, * *p* < 0.05). Furthermore, the content of free BAs, including DCA, LCA, HDCA, and ωMCA, exhibited an increasing trend in the colon and a decreasing trend in the serum. Conversely, the levels of conjugated BAs such as TCA, TUDCA, THDCA, GCA, GLCA, and GHDCA demonstrated a downward trend in the colon, with fecal trends opposite to those in serum (*n* = 8, ** *p* < 0.01, * *p* < 0.05) (Figure 3B).

### 2.4. Effects of HIS on FXR Expression in the Colonic Tissue of Rats with VH

**Figure 4:**
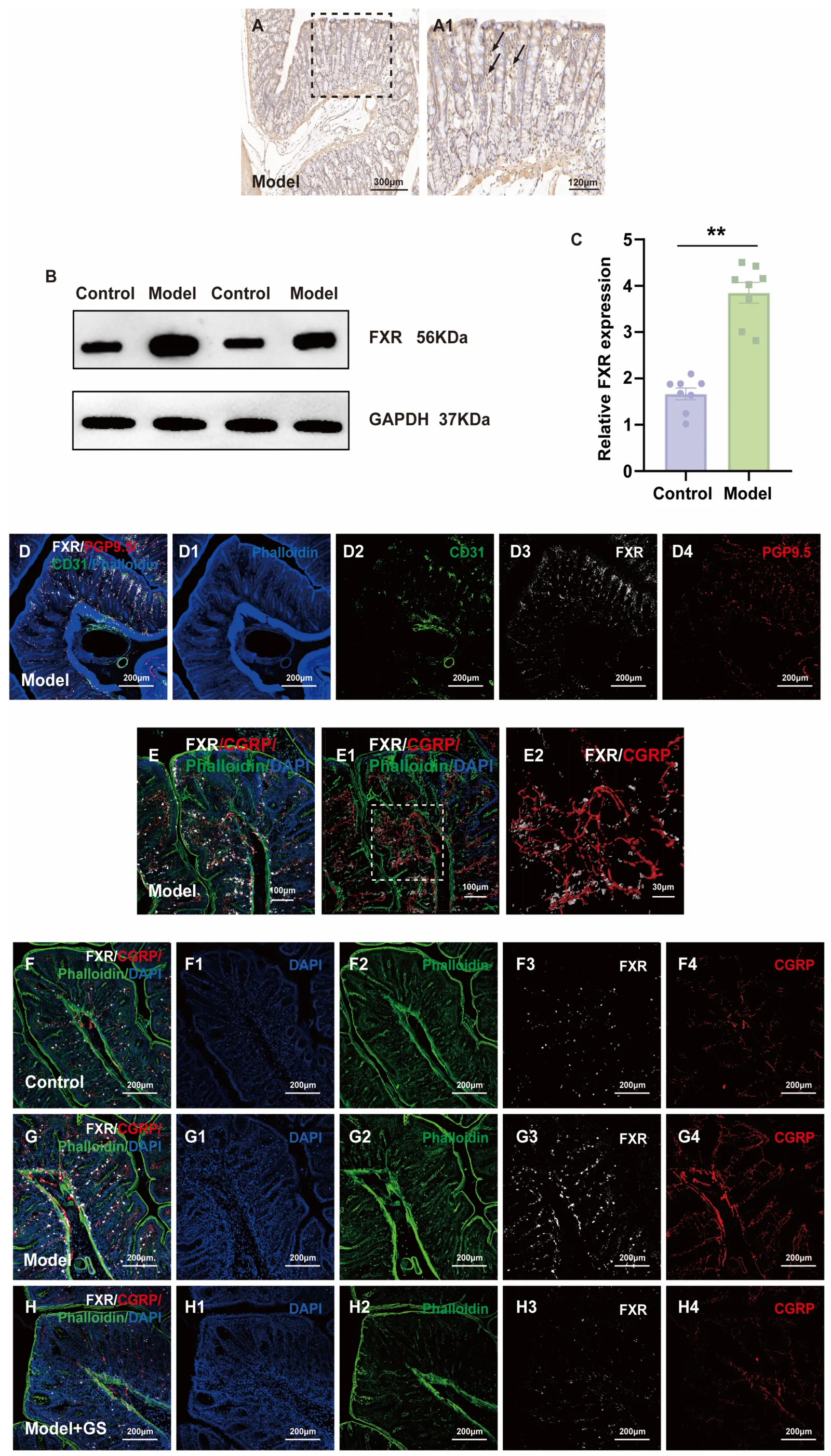
Effects of HIS on colonic FXR expression in IBS rats. (**A**) Representative IHC images showing FXR localization in the colon of model rats; (**A1**) is a high-magnification of the boxed region in (**A**), with arrows indicating FXR-positive signals around the colonic crypts. Scale bars: 300 µm (**A**) and 120 µm (**A1**). (**B**) Representative WB images; GAPDH served as the loading control. (**C**) quantitative analysis of colonic FXR protein expression. (**D**) Merged IF image showing FXR (white), PGP9.5 (red), CD31 (green), and Phalloidin (blue) in the colon of model group rats. showing individual channels for Phalloidin (**D1**), CD31 (**D2**), FXR (**D3**) and PGP9.5 (**D4**). Scale bar = 200 µm. (**E**–**E2**) Representative IF image of the model group colon co-stained for FXR (white), CGRP (red), Phalloidin (green), and DAPI (blue) (**E**); (**E1**) shows the three-dimensional reconstruction of rendered fluorescence signals, and (**E2**) is a magnified view of the boxed region in (**E1**) showing only the FXR and CGRP channels, illustrating the close spatial distribution between CGRP-positive nerve fibers and FXR signals. Scale bars: 100 µm (**E1**) and 30 µm (**E2**). (**F**–**H**) Representative IF merged staining images of colonic tissues from control (**F**), model (**G**), and guggulsterone-treated model (Model + GS) (**H**) rats, showing individual channels for DAPI (**F1**–**H1**), Phalloidin (**F2**–**H2**), FXR (**F3**–**H3**), and CGRP (**F4**–**H4**). Scale bar = 200 µm (*n* = 3). (**C**) Data are expressed as mean ± SEM (*n* = 8). ** *p* < 0.01.

Immunohistochemical (IHC) staining revealed that FXR was predominantly localized around the colonic crypts (Figure 4A). Western blot (WB) analysis further demonstrated that FXR protein expression in the colon was significantly upregulated in the model group compared with the control group (*n* = 8; ** *p* < 0.01; Figure 4B,C), indicating that HIS intervention enhanced colonic FXR expression. Results from multiplex IF staining showed that, in the colon tissue of rats in the model group, FXR and PGP9.5-positive nerve fibers were highly adjacent in their spatial distribution, suggesting a possible close functional connection between FXR and the enteric nervous system (Figure 4D–D4). Three-dimensional reconstruction of the rendered fluorescence signals (Figure 4E,E1) and magnification of the selected region (Figure 4E2) showed a close spatial distribution between CGRP-positive nerve fibers and FXR signals, suggesting a potential interaction between FXR and CGRP-mediated sensory nerves in the colonic tissue of IBS rats. Furthermore, multiplex IF staining of FXR, CGRP, Phalloidin, and DAPI showed stronger FXR and CGRP signals in the model group than in the control group, which were markedly attenuated following treatment with the FXR antagonist guggulsterone (GS) (Figure 4F–H).

## 3. Discussion

Stress is defined as the physiological and behavioral response of an organism to an imbalance of internal and external challenges [17], plays a crucial role in maintaining homeostasis [18]. Chronic stress not only suppresses appetite and causes weight loss [19], but also affects the metabolism of the human body, disrupts endocrine, immune, and nervous system functions [20]. It is a critical factor contributing to VH [21] and alterations in the BA pool [22]. Our findings demonstrate that HIS can induce VH in rats without causing organic colon lesions, consistent with the clinical features of IBS. HIS can lead to weight loss in rats, the resulting suppression of food intake may be closely associated with a modulation of the BA metabolic profile and alterations in the efficiency of lipid and energy absorption. Furthermore, HIS results show the BA pool composition region-specific alterations in feces, colon, and serum and upregulated FXR [23] and CGRP [24] expression in colonic tissues. Notably, three-dimensional confocal reconstruction revealed a close spatial distribution between FXR signals and CGRP-positive nerve fibers, suggesting a potential interplay between the BA receptor and sensory innervation. In combination, these findings indicate that HIS may contribute to the onset of VH by modulating BA metabolism and FXR expression.

Stress is a significant risk factor for metabolic disorders [25]. Chronic exposure to stress conditions can result in the dysfunction of various energy metabolism pathways, including those involving glucose [26] and lipids [27]. Which subsequently affects the BA metabolism and enterohepatic recirculation within the body. BAs, which are the end products of cholesterol metabolism, are crucial for lipid absorption and digestion [28], and alterations in their concentrations can irritate the gastrointestinal tract, provoke inflammatory responses, and contribute to hyperalgesia [29]. From the methodological perspective, the quantitative analysis of BA has traditionally been done by thin-layer chromatography (TLC) [30] and gas chromatography-mass spectrometry (GC-MS) [31]; however, HPLC-MS/MS has emerged as the pivotal analytical platform method owing to its superior sensitivity and high-throughput capacity [32,33]. In this study, we established a comprehensive HPLC-MS/MS method for the quantification of BAs in rat feces, colon, and serum tissues. The research indicates that in model rats the free and conjugated BAs have undergone organ-specific remodeling, which collectively supports the conclusion of disruption of BA homeostasis in the gut-liver axis. The maintenance of BA metabolic homeostasis is highly dependent on the functional regulation of the gut microbiota: bacterial bile salt hydrolases (BSHs) mediate the deconjugation of conjugated BAs, while specific enzyme-producing bacterial groups, represented by the genus Clostridium, catalyze 7α-dehydroxylation to produce the secondary bile acids DCA and lithocholic acid (LCA) [34]. Chronic stress is well known to alter gut microbiota composition [35], raising the possibility that the HIS-induced BA alterations observed in this study are partly or entirely mediated by stress-induced changes in the gut microbiota. Without 16S rRNA sequencing or metagenomic data, we cannot distinguish whether HIS directly alters host BA synthesis and conjugation, whether HIS-induced changes in the microbiota secondarily drive the observed BA remodeling, or whether both mechanisms operate in parallel. Future studies integrating microbiota profiling with fecal microbiota transplantation or antibiotic-mediated depletion will be essential to establish the causal contribution of the gut microbiota to the BA–FXR axis. In addition, our compositional analysis further revealed species-specific changes: fecal levels of HDCA and GHDCA increased, while ωMCA, TUDCA, and THDCA decreased; colonic DCA, LCA, and ωMCA were increased, whereas GCA and GLCA declined; and serum TCA and TUDCA increased while LCA and GLCA decreased. These specific alterations may be linked to the stress-induced dysfunction section and the development of VH. The above differences are physiologically important because individual BA species differ substantially in their receptor affinity profiles. FXR is activated most potently by CDCA, followed by DCA, LCA, and CA, whereas UDCA can inhibit FXR [36]; in contrast, the receptor TGR5 displays a preference for secondary BAs, with the potency order LCA > DCA > CDCA > CA [37]. Thus, the observed remodeling of the BA pool may reshape the relative engagement of FXR or TGR5 signaling within the colonic mucosa, with downstream consequences for epithelial barrier integrity, immune tone, and afferent nerve excitability.

In this exploratory study, we focused on FXR because it is highly expressed in the colon, centrally regulates BA homeostasis and intestinal barrier function, and our data demonstrated colonic FXR upregulation with close spatial association to CGRP-positive nerve fibers, which was attenuated by the FXR antagonist guggulsterone. However, we acknowledge that several altered species are TGR5 ligands, and TGR5 was not directly assessed here. Therefore, our findings should be interpreted as identifying the BA–FXR axis as a candidate pathway, while the potential contribution of TGR5 and other BA receptors warrants further investigation using pathway-specific agonists/antagonists and receptor expression analyses.

FXR serves as a pivotal regulatory factor in the adaptive response to BA, significantly influencing physiological processes within the organism [38]. As a nuclear receptor for BAs, FXR modulates the levels of BAs in the body through a negative feedback mechanism [39] and maintains intestinal homeostasis [40]. FXR exhibits varying affinities for different BA species, with chenodeoxycholic acid (CDCA), deoxycholic acid (DCA), lithocholic acid (LCA) and their conjugated forms acting as effective agonists [41]. Activation of intestinal FXR inhibits BA synthesis and downregulates CYP8B1 expression, which can reduce intestinal pain [42]. Our study’s findings indicate that following intervention with HIS, there was a significant increase in the concentrations of colonic LCA and DCA, accompanied by a notable decrease in the levels of glycocholic acid (GCA) and glycolithocholic acid (GLCA), as well as a marked upregulation of colonic FXR protein expression. These alterations in BA metabolism and FXR expression suggest that the BA–FXR axis may be involved in stress-induced visceral hypersensitivity.

To explore whether FXR signaling contributes functionally to these changes, we employed the FXR antagonist guggulsterone. Guggulsterone administration attenuated both FXR and CGRP signals, providing pharmacological evidence consistent with the involvement of FXR activation in stress-induced sensory changes. Because guggulsterone treatment began before the first stress exposure, any protective effect would reflect prevention during model induction rather than reversal of established hypersensitivity. These results imply that HIS may induce VH through the modulation of BA metabolism and FXR signaling. However, it should be noted that guggulsterone is a pleiotropic compound with documented effects beyond FXR antagonism, including inhibition of NF-κB, AP-1, and STAT3 signaling and modulation of steroid hormone receptors [43]. The attenuation of FXR and CGRP signals observed in this study may therefore not be attributable exclusively to FXR antagonism. Given this limitation, our guggulsterone data should be interpreted as suggestive rather than definitive evidence for FXR’s role; confirmation using FXR-specific genetic approaches or more selective FXR antagonists will be required in future studies. We did not perform a formal dose–response analysis or directly measure guggulsterone concentrations in plasma and colonic tissue; although suppression of colonic FXR target genes confirms target engagement, future studies including multiple doses and tissue pharmacokinetics will be needed to define the optimal dosing regimen.

Previous mechanistic studies of VH in IBS have primarily focused on neuro-endocrine interactions [44]; however, the emergence of VH is not attributable to nervous pain transmission but is also intricately linked to disruptions in internal homeostasis, among which dysregulated BA homeostasis is a significant contributing factor [45]. Chronic stress-induced VH is now recognized as a multifactorial phenomenon: in addition to the BA metabolic alterations observed in our study, previous studies have implicated dysregulation of the CRF–ACTH–corticosterone/cortisol rhythm, enhanced sympathetic tone with reduced vagal activity, and increased central gain with weakened descending inhibition, involving the amygdala, insula, anterior cingulate cortex, hippocampus, prefrontal cortex, periaqueductal gray, and rostral ventromedial medulla; imbalances in descending noradrenergic, serotonergic, and endogenous opioid inhibition, together with pain anticipation, anxiety, and rumination, further amplify visceral sensitivity [21]. BA–FXR signaling should therefore be regarded as one contributing factor rather than the sole mechanism. Li WT et al. demonstrated that BA activate the FXR, inducing the release of nerve growth factor (NGF) and transient receptor potential vanilloid 1 (TRPV1) through mast cells, thereby contributing to the development of colonic VH [23]. Our study corroborates and extends these findings by spatially resolving the juxtaposition of FXR and CGRP-positive nerve fibers through three-dimensional confocal reconstruction. Because FXR is expressed in multiple colonic cell types, including enterocytes, enteroendocrine cells, and immune cells such as macrophages and dendritic cells [46], we additionally performed double-label IF staining for the pan-neuronal marker PGP9.5 and FXR in colonic tissue after modeling to clarify whether FXR is expressed within neuronal structures or in adjacent cell types. The current data cannot fully exclude FXR expression in enterocytes, enteroendocrine cells, or immune cells, and future studies employing additional neuronal markers (e.g., NF200 or peripherin) or single-cell approaches will be needed to define the cell-type-specific contribution of FXR to visceral sensation. Further, we will focus on elucidating the BA–FXR signaling cascade at the molecular and cellular levels, which may inform the development of FXR-targeted therapeutic strategies for IBS.

Several limitations of the present study should be acknowledged. First, with respect to model and design, only male rats were employed to minimize the confounding effects of estrous cycle fluctuations, yet IBS exhibits a well-documented female predominance, with women demonstrating heightened visceral perception and differential stress reactivity compared to men [47]; moreover, no sham-stressed control group was included, so we cannot fully exclude the possibility that routine handling contributed to the observed changes. Second, with respect to phenotyping and sampling, comprehensive metabolic characterization (physical activity, energy expenditure, body composition) was not performed, and colonoscopy with distilled-water irrigation preceded terminal tissue collection, which even though limited in volume and temporally remote could transiently have altered the colonic BA pool. Third, with respect to mechanistic interpretation, VH was not functionally assessed in the guggulsterone-treated group, so the reduced FXR and CGRP immunoreactivity represents correlative rather than functional evidence; guggulsterone itself is pleiotropic and no response or tissue pharmacokinetic data were obtained, such that FXR involvement requires confirmation using FXR-specific genetic approaches or more selective antagonists; TGR5 and other BA receptors were not directly assessed; the spatial association between FXR and CGRP-positive fibers is qualitative, as reliable quantitative colocalization analysis could not be performed; and the absence of 16S rRNA sequencing or metagenomic data prevents determination of whether the BA remodeling is mediated by the gut microbiota rather than by a direct host effect. Accordingly, future studies should incorporate female rats across estrous stages, a fully matched sham-stressed control, functional VH testing upon FXR inhibition, pathway-specific receptor analyses, microbiota sequencing with depletion or transplantation experiments, quantitative colocalization analysis, and pre-endoscopic tissue collection to confirm that the BA–FXR–CGRP alterations are specifically driven by HIS and to systematically validate the therapeutic potential of this axis for VH.

## 4. Materials and Methods

### 4.1. Animals

Adult male Sprague-Dawley rats, sourced from SPF (Beijing) Biotechnology Co. Ltd., Beijing, China, were randomly allocated into control and model groups, with each rat weighing 206.5 ± 1.1 g. The experiment was conducted under controlled laboratory conditions, maintaining an ambient temperature of 22–24 °C and a 12 h light–dark cycle. The rats had ad libitum access to food and water. All experiments were completed in three separate experiments. In the first experiment, 16 rats were randomly divided into a control group and a model group (*n* = 8 per group); these animals were monitored for body weight changes and underwent colonoscopy and abdominal EMG recordings; after completing the EMG recordings, colon tissue was harvested via cardiac perfusion with a 4% paraformaldehyde solution while the rats were under deep anesthesia for HE, IF, and IHC analysis. In the second experiment, 16 rats were randomly divided into a control group and a model group (*n* = 8 in each group); these animals were used to collect fecal, serum, and colonic samples for HPLC-MS/MS bile acid metabolomics analysis and Western blotting (*n* = 8 in each group). In the third experiment, 9 rats were randomly divided into a control group, a model group, and a gugulsterone treatment group (*n* = 3 per group), and colon tissue was collected for IF analysis. The control animals were housed under the same conditions and underwent the same experimental procedures and handling as the model group, including colonoscopy and colorectal distension/EMG procedures where applicable, but were not exposed to the heterotypic intermittent stressors, thereby serving as sham-stressed controls. The study received approval from the Animal Ethics Committee of the Institute of Acupuncture and Moxibustion, China Academy of Chinese Medical Sciences (Approval No. D2024-01-26-05). All procedures complied with the National Research Council’s Guide for the Care and Use of Laboratory Animals, and strict adherence to the 3R principles (Replacement, Reduction, and Refinement) was ensured throughout the experiment.

### 4.2. HIS Modeling Approach

Rats were subjected to a high-intensity stress regimen for nine consecutive days, with one of three stressors administered daily between 8:00 and 11:00 a.m.: cold restraint stress (CRS), ice water stress (IWS), or forced swimming stress (FSS). For CRS, rats were confined in metal cages and placed in a cold chamber at 4 °C for 2 h (days 1, 6, and 8). For IWS, rats were placed in a plastic container (20 cm in height, 10 cm in width) filled with ice water at 4 °C for approximately 7 min (days 2, 4, and 9). For FSS, rats were forced to swim in a plastic container (40 cm × 20 cm × 20 cm). The water was poured into the container up to 17 cm below the top, with a temperature of about 22 °C for 7 min (days 3, 5, and 7) [48]. During all cold and swimming procedures, animals were continuously monitored for signs of labored breathing, cyanosis, loss of balance, or severe distress. If any of these signs appeared, the procedure was immediately terminated, and the animal was transferred to a warm recovery cage. After each stress session, rats were gently dried with a towel and placed in a heated cage (30–32 °C) for 30 min to recover before returning to their home cages. Humane endpoints were strictly applied throughout the study.

### 4.3. FXR Antagonist Administration

To investigate the role of FXR in HIS-induced VH, rats received the FXR antagonist guggulsterone (S3792, Selleck Chemicals, Houston, TX, USA) by oral gavage at 40 mg/kg body weight once daily. A stock solution of guggulsterone was prepared in a mixed solvent of 5% DMSO (D8370, Solarbio, Beijing, China), 40% PEG300 (S6704, Selleck Chemicals, Houston, TX, USA), 5% Tween-80 (T8360, Solarbio, Beijing, China), and 50% purified water, and diluted with purified water immediately before use so that each rat received a daily gavage volume of 1 mL. The drug was administered once daily prior to stress exposure, starting on the first day of the HIS and continuing throughout the entire 9-day stress period [49,50,51].

### 4.4. EMG Recording of VH During Colorectal Distension

VH was evaluated by EMG recording of the visceromotor response (VMR) during CRD [20]. EMG recordings were performed 24 h after completion of the final HIS stressor to avoid residual acute stress effects. As previously outlined in the study [20], rats were anesthetized with isoflurane (R510-22, RWD Life Science, Shenzhen, China) (induction concentration 3–4%, maintenance concentration 1.5–2%). Needle electrodes were implanted into the external oblique abdominal muscle and connected to a PowerLab signal acquisition and analysis system (PL3516, AD Instruments, Dunedin, New Zealand) to monitor electromyographic changes. A balloon measuring 4–5 cm was created using the finger of a medical glove fitted with a connecting tube; this was then inserted into the colon via the anus to a depth of 6–8 cm and held in place by securing the tube to the tail. The balloon was calibrated using a sphygmomanometer connected to a pressure transducer before each experiment. Following a 30 min acclimatization period, the balloon was rapidly increased at a consistent stationary frequency to pressures of 20, 40, 60, and 80 mmHg, in a fixed ascending order, each maintained for 30 s with a 3 min recovery interval between distensions. The EMG signal was amplified, filtered, and digitized at a sampling rate of 1 kHz. Baseline activity was recorded for 30 s before each distension and subtracted from the distension period. The area under the curve (AUC) of the EMG was calculated for each 30 s distension period using Spike2 software (version 8.0, Cambridge Electronic Design Ltd., Cambridge, UK), representing the increment above baseline. Each rat underwent two repeated EMG assessments at each distension pressure, and the mean of the two repeated measurements was used for statistical analysis of the abdominal external oblique muscle EMG activity [11].

### 4.5. Colonoscopy Procedures

Following the administration of isoflurane anesthesia in rats, the endoscope (7218AA, Karl Storz Veterinary Endoscopy-America, Inc., Goleta, CA, USA) was meticulously inserted transanally under direct visualization. It is imperative to confirm the presence of any abnormal lesions in the colonic tissue using the endoscope once the colon has been properly identified. Subsequently, a volume of 1–2 mL of distilled water was employed to irrigate the intestine. After each procedure on different specimens, the endoscope was extracted and rigorously cleansed with 75% ethanol to ensure sterility and minimize potential confounding of colonic BA measurements by this procedure.

### 4.6. Sample Collection

All samples were collected within 24 h after the final stressor, between 8:00 and 11:00 a.m. to minimize circadian variation. Rats were deeply anesthetized with isoflurane and euthanized by exsanguination under deep anesthesia. Blood was collected via cardiac puncture immediately after confirmation of deep anesthesia, and serum was obtained by centrifugation at 3000× *g* for 10 min at 4 °C, then aliquoted, snap-frozen in liquid nitrogen, and stored at −80 °C until analysis.

For BAs quantification, feces were collected from the anus immediately after sacrifice. Approximately 4 cm of colon tissue was rapidly excised, snap-frozen in liquid nitrogen, and stored at −80 °C for BAs quantification, Western blot analysis, and metabolomics. Colon tissues for histological examination were obtained from animals in the first and the three experiments after transcardial perfusion with 0.9% saline followed by 4% paraformaldehyde under deep anesthesia to fix and then stored at −80 °C for histological examination.

### 4.7. HE, IF and IHC Staining

#### 4.7.1. HE

Rats were deeply anesthetized with isoflurane and transcardially perfused with 0.9% normal saline, followed by fixation with 4% paraformaldehyde solution. Colonic tissues were collected, post-fixed in 4% paraformaldehyde for 24 h, embedded in paraffin, and sectioned at a thickness of 5 μm. The sections were then stained with hematoxylin and eosin according to standard protocols.

#### 4.7.2. IF

Tissue sections were cut into 35 μm thick slices using a cryostat (CM1950, Leica Biosystems Nussloch GmbH, Nussloch, Germany). The cryosections were placed in a solution containing 0.5% Triton X-100 (T8787, Sigma-Aldrich, St. Louis, MO, USA) and 3% donkey serum (017-000-121, Jackson ImmunoResearch Laboratories Inc., West Grove, PA, USA) and underwent permeabilization and blocking at 37 °C for 30 min. Subsequently, the sections were incubated overnight at 4 °C with primary antibodies diluted in 0.1 M phosphate-buffered saline (PBS) containing 0.5% Triton X-100: mouse anti-CGRP monoclonal antibody (1:500; ab81887, Abcam, Cambridge, UK) and rabbit anti-FXR polyclonal antibody (1:500; A24015, ABclonal Technology, Wuhan, China) (Experiment 1)/rabbit anti-FXR polyclonal antibody (1:500; A24015), mouse anti-PGP9.5 monoclonal antibody (1:200; ab8189, Abcam, Cambridge, UK), and goat anti-CD31 polyclonal antibody (1:500; AF3628, Novus Biologicals, Centennial, CO, USA) (Experiment 3). After washing three times with phosphate-buffered saline (PBS), the sections were incubated at room temperature with fluorescently labeled phalloidin (1:500; A12379, Life Technologies, Carlsbad, CA, USA), Alexa Fluor 647-labeled donkey anti-rabbit IgG (1:500; A-31573, Thermo Fisher Scientific, Waltham, MA, USA), Alexa Fluor 568-labeled donkey anti-mouse IgG (1:500; A10037, Thermo Fisher Scientific, Waltham, MA, USA) (Experiment 1); and Alexa Fluor 488-labeled donkey anti-goat IgG (1:500; A16009, Thermo Fisher Scientific, Waltham, MA, USA) (Experiment 3). All of the above antibodies were diluted in 0.1 M PBS containing 0.5% Triton X-100. Finally, cell nuclei were counterstained with 4′,6-diamino-2-phenylindole (DAPI) (S2110, Solarbio, Beijing, China), the sections were mounted with an anti-fade mountant, and images were acquired using a confocal laser scanning microscope (AX, Nikon Corporation, Tokyo, Japan). Z-stack images of colonic sections multiplexed for FXR, CGRP, phalloidin, and DAPI were acquired using a confocal laser scanning microscope with a 20× objective at a step size of 1 μm. Three-dimensional reconstruction and rendering of the fluorescence signals were performed using Imaris software (version 10.0, Oxford Instruments, Abingdon, UK).

The spatial relationship between CGRP-positive nerve fibers and FXR signals was examined in the rendered three-dimensional images and magnified views of selected regions. To assess nonspecific binding, negative control sections were processed identically but incubated without primary antibodies, and only background staining was observed.

#### 4.7.3. IHC

Paraffin-embedded tissue sections (4 μm) were dewaxed in xylene and rehydrated through a graded series of ethanol. After 10 min of high-temperature heat-induced antigen retrieval using a citrate buffer, the sample was cooled at room temperature for 20 min. Endogenous peroxidase activity was blocked by incubating with 3% hydrogen peroxide solution for 10 min at room temperature. After blocking with normal serum for 20 min, the sections were sequentially incubated with primary and secondary antibodies, visualized with DAB, and counterstained with hematoxylin. Finally, the sections were dehydrated, cleared, mounted, and observed under a microscope (AX, Nikon Corporation, Tokyo, Japan).

### 4.8. HPLC-MS/MS for Detection of BA Metabolites

The samples’ pretreatment and quantitative analyses were carried out using the method described [52]. In brief, 100 µL of serum were mixed with 400 µL of pre-cooled (–20 °C) methanol, vortexed for 5 min., and subsequently centrifuged at 20,000× *g* for 10 min at 4 °C. 180 µL of supernatant were collected and 20 µL of the bile acid internal standard mixture (10 µg/mL [2H4]-cholic acid and [2H4]-taurocholic acid) were added. The samples were again shaken for 1 min and centrifuged as previously described and the supernatant were collected for analysis. To extract metabolites from feces and colon tissue sampes, frozen samples were ground with liquid nitrogen. The resultant feces and colon tissue powder (~100 mg) was weighed and then extracted with 4000 µL extraction solvent (methanol:water = 80:20, *v*/*v*, pre-cooled at –20 °C), homogenized for 20 s with a biological sample homogenizer (Precellys Evolution), ultrasonicated for 10 min, and subsequently centrifuged at 20,000× *g* for 10 min at 4 °C. 180 µL of supernatant was collected, and 20 µL of the bile acid internal standard mixture (as described above) was added. After shaken for 1 min, the samples were centrifuged again as described above. The supernatant was collected for analysis.

For quantitative analyses, an HPLC-MS/MS system (AB Sciex, Framingham, MA, USA) was performed. The separation was performed on a BEH C18 column (2.1 mm × 100 mm, 1.7 μm, Waters Corp., Milford, MA, USA). The mobile phase consisted of two solvents: an aqueous solution with 0.1% formic acid (A) and acetonitrile with 0.1% formic acid (B). Gradient elution was performed for 25.0 min at a flow rate of 0.3 mL/min. The gradient conditions were as follows: 0–1.0 min, 20% B–25% B; 1.0–5.0 min, 25% B; 5.0–7.0 min, 25% B–30% B; 7.0–8.0 min, 30% B; 8.0–11.0 min, 30% B–40% B; 11.0–13.0 min, 40% B; 13.0–19.0 min, 40% B–70% B; 19.0–20.0 min, 70% B–95% B; 20.0–22.5 min, 95% B; 22.5–22.51 min, 95% B–20% B; 22.51–25.0 min, 20% B. 5 µL aliquots of each sample were injected into the chromatographic column. Temperatures in the column were maintained at 50 °C. Analysis of all samples was carried out at 4 °C.

Mass spectrometry was carried out by a Sciex 6500+ triple quadrupole mass spectrometer equipped with a Turbo V ion source. All components have been detected in negative electrospray ion mode. Curtain gas, collision gas, nebulizer gas (GS1), and turbo gas (GS2) have been set at 35, 9, 50, and 50 psi, respectively. The electrospray voltage was set at 4500 V, and the turbo ion spray source temperature was 550 °C. Nitrogen was used as the collision gas. The system operated in multiple reaction monitoring (MRM) mode. The quantitative transitions and their dwell time, collision energy, declustering potential, and retention time are shown in Table 2. Prepare a series of standards with known concentrations (range: 1–5000 ng/mL), and extract concentrations were calculated using the corresponding calibration curves. [2H4]-Cholic acid and [2H4]-taurocholic acid were used as the internal standards for free and conjugated bile acids, respectively. Data acquisitions were performed using Analyst 1.7.1 software (AB Sciex, Framingham, MA, USA). The Multiquant 3.0.3 software (AB Sciex, Framingham, MA, USA) was used to quantify all analytes.

### 4.9. WB Analysis

Colon tissues were homogenized in ice-cold RIPA buffer with PMSF (100:1) and centrifuged at 14,000 rpm for 20 min at 4 °C. Protein concentrations in the supernatants were determined using a BCA assay kit (AR0146, Wuhan Boster Biological Technology Co., Ltd., Wuhan, China). Equal amounts of protein were denatured, separated by SDS-PAGE, and transferred onto PVDF membranes. After blocking with 5% nonfat milk in TBST for 1 h at room temperature, the membranes were incubated overnight at 4 °C with rabbit polyclonal anti-FXR (1:500; bs-12867R, Bioss, Beijing, China) or rabbit anti-GAPDH (1:3000; 10494-1-AP, Proteintech Group Inc., Wuhan, China) antibodies, followed by incubation with HRP-conjugated goat anti-rabbit IgG (1:10,000; RS0002, ImmunoWay Biotechnology, Plano, TX, USA) for 70 min at room temperature. Protein bands were visualized by enhanced chemiluminescence (ECL) and quantified using ImageJ software (v1.54, National Institutes of Health, Bethesda, MD, USA), with GAPDH serving as the internal reference for normalization.

### 4.10. Statistical Analysis

The data were subjected to statistical analysis utilizing SPSS software version 26.0 (IBM, Armonk, NY, USA). Results are presented as mean ± standard error of the mean (SEM). For comparisons between two groups, either Student’s *t*-test or a Mann–Whitney U test was employed. Unless otherwise specified, *p*-values less than 0.05 were deemed statistically significant.

## 5. Conclusions

This study demonstrates that HIS induces VH in rats without organic colon lesions, accompanied by enhanced CGRP-mediated nerve fibers. Using an HPLC-MS/MS testing technique for multi-site BA profiling in VH rats, the results show that HIS remodels the BA pool composition in feces, colon, and serum in a region-specific manner: the free-to-conjugated BA ratio was significantly increased in the colon and decreased in the serum, whereas no significant difference was detected in feces. HIS also significantly upregulated colonic FXR expression. Combined with three-dimensional confocal reconstruction technology, the observed close spatial distribution of FXR with CGRP-positive nerve fibers, and the weaker of both signals by the FXR antagonist guggulsterone indicate that the BA–FXR axis contributes to stress-induced VH, most likely through modulation of colonic sensory nerve innervation. The BA–FXR axis, interacting with sensory innervation, may prove to be a therapeutic target for IBS. However, given that guggulsterone is a multi-targeted compound, this pharmacological evidence should be interpreted as suggestive rather than definitive proof of FXR involvement. Future research will still focus on mechanism studies at the cellular and molecular levels to further elucidate the causal relationships within this pathway and explore its translational potential.

## Figures and Tables

**Table 1:** The content and proportion of free and conjugated BAs.

	BAs	Control	Fraction	Model	Fraction	Fraction Ratio *p*
Fecal	Total	1,048,193.27 ± 174,469.18		799,236.40 ± 56,895.87		NS
	Free	1,047,117.20 ± 174,233.26	99.91% ± 0.01%	798,632.12 ± 56,891.04	99.92% ± 0.01%	
	Conjugated	1076.07 ± 262.85	0.09% ± 0.01%	604.29 ± 78.15	0.08% ± 0.01%	
	Ratio	1271.09 ± 261.39		1463.95 ± 185.60		
Colon	Total	26,208.64 ± 3719.38		37,183.71 ± 5196.71		NS
	Free	25,960.65 ± 3676.99	99.08% ± 0.04%	36,991.30 ± 5171.29	99.48% ± 0.01%	**
	Conjugated	248.00 ± 42.78	0.92% ± 0.04%	192.42 ± 26.01	0.52% ± 0.01%	**
	Ratio	108.98 ± 4.06		193.11 ± 5.33		**
Serum	Total	7351.34 ± 1110.30		6103.83 ± 1040.76		NS
	Free	6837.74 ± 1026.63	93.11% ± 0.20%	5434.80 ± 934.69	88.97% ± 0.42%	**
	Conjugated	513.61 ± 85.06	6.89% ± 0.20%	669.04 ± 110.05	11.03% ± 0.42%	**
	Ratio	13.60 ± 0.42		8.17 ± 0.36		**

Content and proportion of free and conjugated BAs in the feces, colon, and serum of control and model rats. Among the 27 endogenous BAs initially screened, 24 were included in the final composition analysis; three tauro-muricholic acids (T-α-MCA, T-β-MCA, and T-ω-MCA) were excluded due to insufficient detection. (Data are expressed as mean ± SEM. (*n* = 8); NS > 0.05; ** *p* < 0.01.

**Table 2:** Mass spectrometer setup for the quantification of selected BAs.

Analytes	Abbreviation	MRM (Transition)	Declustering Potential (V)	Collision Energy (eV)	Retention Time (min)
cholic acid	CA	407.4/407.4	−150	−5	15.00
chenodeoxycholic acid	CDCA	391.4/391.4	−150	−5	18.00
ursodeoxycholic acid	UDCA	391.4/391.4	−150	−5	15.31
hyodeoxycholic acid	HDCA	391.4/391.4	−150	−5	15.55
deoxycholic acid	DCA	391.4/391.4	−150	−5	18.31
lithocholic acid	LCA	375.1/375.1	−150	−5	20.68
hyocholic acid	HCA	407.2/407.2	−150	−5	13.78
ursocholic acid	UCA	407.2/407.2	−150	−5	10.79
dehydrocholic acid	DHCA	401.4/401.4	−150	−5	11.00
α-muricholic acid	α-MCA	407.2/407.2	−150	−5	12.45
β-muricholic acid	β-MCA	407.2/407.2	−150	−5	12.78
ω-muricholic acid	ω-MCA	407.2/407.2	−150	−5	12.13
taurocholic acid	TCA	514.5/80.1	−150	−120	10.37
taurochenodeoxycholic acid	TCDCA	498.4/80.1	−150	−120	12.60
tauroursodeoxycholic acid	TUDCA	498.4/80.1	−150	−120	9.67
taurohyodeoxycholic acid	THDCA	498.4/80.1	−150	−120	9.84
taurodeoxycholic acid	TDCA	498.4/80.1	−150	−120	12.78
taurolithocholic acid	TLCA	482.3/80.1	−150	−120	16.42
tauro-α-muricholic acid	T-α-MCA	514.3/80.1	−150	−120	5.91
tauro-β-muricholic acid	T-β-MCA	514.3/80.1	−150	−120	5.69
tauro-ω-muricholic acid	T-ω-MCA	514.3/80.1	−150	−120	5.30
glycocholic acid	GCA	464.4/74.1	−150	−70	12.45
glycochenodeoxycholic acid	GCDCA	448.4/74.1	−150	−70	15.62
glycoursodeoxycholic acid	GUDCA	448.4/74.1	−150	−70	12.26
glycohyodeoxycholic acid	GHDCA	448.4/74.1	−150	−70	12.39
glycodeoxycholic acid	GDCA	448.4/74.1	−150	−70	16.19
glycolithocholic acid	GLCA	432.5/74.1	−150	−70	18.59
[2H4]-cholic acid	D4-CA	411.4/411.4	−150	−5	15.00
[2H4]-taurocholic acid	D4-TCA	518.5/80.1	−150	−120	10.33

## Data Availability

This manuscript contains all data generated or analyzed in this study. For further inquiries, please contact the corresponding authors.

## Abbreviations

The following abbreviations are used in this manuscript:

AUC	Area under the curve
BA	Bile Acid
CDCA	Chenodeoxycholic acid
CGRP	Calcitonin gene-related peptide
CRD	Colorectal distension
CRS	Cold restraint stress
DCA	Deoxycholic acid
EMG	Electromyographic
FSS	Forced swimming stress
FXR	Farnesoid X receptor
GCA	Glycocholic acid
GHDCA	Glycohyodeoxycholic acid
GLCA	Glycolithocholic acid
HDCA	Hyodeoxycholic acid
HE	Hematoxylin-eosin
HIS	Heterotypic intermittent stress
HPLC-MS/MS	High-performance liquid chromatography–mass spectrometry
IBS	Irritable bowel syndrome
IF	Immunofluorescence
IHC	Immunohistochemistry
IWS	Ice water stress
LCA	Lithocholic acid
PCA	Principal component analysis
TCA	Taurocholic acid
THDCA	Taurohyodeoxycholic acid
TUDCA	Tauroursodeoxycholic acid
VH	Visceral hypersensitivity
WB	Western blot
ωMCA	ω-Muricholic acid
